# *ATM* polymorphisms as risk factors for prostate cancer development

**DOI:** 10.1038/sj.bjc.6602007

**Published:** 2004-07-27

**Authors:** S Angèle, A Falconer, S M Edwards, T Dörk, M Bremer, N Moullan, B Chapot, K Muir, R Houlston, A R Norman, S Bullock, Q Hope, J Meitz, D Dearnaley, A Dowe, C Southgate, A Ardern-Jones, The Cancer Research UK/British Prostate Group/Association of Urological Surgeons, Section of Oncology Collaborators, D F Easton, R A Eeles, J Hall

**Affiliations:** 1 DNA Repair Group, International Agency for Research on Cancer, 150 cours Albert Thomas, 69373 Lyon, France; 2 The Institute of Cancer Research, Sutton, Surrey, UK; 3 Clinics of Obstetrics and Gynaecology, Medical School Hannover, Podbielskistr. 380, D-30659 Hannover, Germany; 4 Department of Radiation Oncology, Medical School Hannover, Carl-Neuberg-Str. 1, D-30625 Hannover, Germany; 5 Department of Epidemiology, University of Nottingham, UK; 6 Royal Marsden NHS Trust, 15 Cotswold Road, Sutton, Surrey SM2 5NG, UK; 7 List available on request; 8 Cancer Research UK, Genetic Epidemiology Unit, Strangeways Research Laboratory, Worts Causeway, Cambridge CB1 8RN, UK

**Keywords:** *ATM*, prostate cancer susceptibility, polymorphisms

## Abstract

The risk of prostate cancer is known to be elevated in carriers of germline mutations in *BRCA2*, and possibly also in carriers of *BRCA1* and *CHEK2* mutations. These genes are components of the *ATM*-dependent DNA damage signalling pathways. To evaluate the hypothesis that variants in *ATM* itself might be associated with prostate cancer risk, we genotyped five *ATM* variants in DNA from 637 prostate cancer patients and 445 controls with no family history of cancer. No significant differences in the frequency of the variant alleles at 5557G>A (D1853N), 5558A>T (D1853V), ivs38-8t>c and ivs38-15g>c were found between the cases and controls. The 3161G (P1054R) variant allele was, however, significantly associated with an increased risk of developing prostate cancer (any G *vs* CC OR 2.13, 95% CI 1.17–3.87, *P*=0.016). A lymphoblastoid cell line carrying both the 3161G and the 2572C (858L) variant in the homozygote state shows a cell cycle progression profile after exposure to ionising radiation that is significantly different to that seen in cell lines carrying a wild-type *ATM* gene. These results provide evidence that the presence of common variants in the *ATM* gene, may confer an altered cellular phenotype, and that the *ATM* 3161C>G variant might be associated with prostate cancer risk.

Prostate cancer is the second most common malignancy and the second commonest cause of cancer deaths in men in the European Union, with 143 000 new cases and 60 000 deaths year^−1^ (GLOBCAN 2000, www-dep.iarc.fr). The aetiology of prostate cancer is poorly understood. Prostate cancer is known to aggregate in families, indicating that genetic susceptibility may be important, but the genes involved are largely unknown. Linkage studies in multiple case families have suggested susceptibility loci on chromosomes 1q24, 1q42, 1p36, 8p22–23, 17p, 20q13 and Xq (see recent reviews by DeMarzo *et al*, 2003; Gronberg, 2003) but none have been definitively replicated. As a consequence of these linkage studies, variants in prostate cancer families have been identified in several genes including Macrophage Scavenger Receptor 1(*MSR1*), 2′-5′-oligoadenylate-dependent ribonuclease L (*RNASEL*) and *ELAC2* (chromosome 17p11/*HPC2* region) (reviewed in Simard *et al*, 2003), but again none have been reliably associated with risk.

Several independent studies have demonstrated that individuals with germline mutations in *BRCA2* are at increased risk of prostate cancer (The Breast Cancer Linkage Consortium, 1999; Edwards *et al*, 2003; Giusti *et al*, 2003). There is also some evidence for an increased risk in carriers of *BRCA1* mutations (Thompson *et al*, 2002). More recently, Seppala *et al* (2003) have found that the CHEK2 variant 1100delC, known to be associated with an increased risk of breast cancer, is also associated with an increased risk of prostate cancer, and Dong *et al* (2003) found that this and other missense variants in CHEK2 occurred at increased frequency in prostate cancer cases. The proteins encoded by the *BRCA1* and *BRCA2* genes participate in the maintenance of genomic stability through their involvement in the homologous recombination pathway for the repair of DNA double-strand breaks and transcription coupled repair and the CHEK2 protein is also involved in DNA damage signalling pathways. BRCA1 and CHEK2 are both phosphorylated in response to DNA damage in an ATM-dependent fashion (Matsuoka *et al*, 2000). Thus, we hypothesised that the *ATM* gene, whose protein functions upstream of these known susceptibility genes, could also be a mutation target in prostate cancer.

In a preliminary study by Hall *et al* (1998), germline mutations in *ATM* were identified in three out of 17 (17.6%) prostate cancer patients who showed a severe late response to radiation therapy and in whom most or all of the *ATM* gene was examined, while no such mutations were found in the control group. In this same study, the 5557G>A *ATM* sequence variant was found in 35% of cases compared with the reported population frequency of 15%. This variant has been found to modulate the penetrance of hereditary nonpolyposis colorectal cancer in carriers of germline *MLH1* and *MLH2* mutations (Maillet *et al*, 2000). Loss of heterozygosity of chromosome 11q, the location of the *ATM* gene, has also been reported in metastatic prostate carcinoma (Ruijter *et al*, 1999).

In order to assess whether *ATM* variants play a pathogenic role in prostate cancer development, we compared the frequencies of five *ATM* single-nucleotide polymorphisms (SNPs) 5557G>A, 5558A>T, 3161C>G, ivs38-8t>c, ivs38-15g>c in 618 British prostate cancer cases and 445 controls. In addition, the cellular phenotype of a lymphoblastoid cell line carrying the 3161G variant allele in a homozygote state was evaluated.

## MATERIALS AND METHODS

### Subjects

Subjects were obtained from a study of prostate cancer cases treated at the Royal Marsden NHS Trust over the period 1993–2002, as previously described (Eeles *et al*, 1999). The patients were unselected for age or family history. The current study included 637 Caucasian cases diagnosed between ages 43 and 86 years.

### Controls

Controls were recruited from two series. Series one comprised of spouses of patients enrolled in a population-based study of colorectal cancer in the UK (84 males, 86 females) Series 2 (*n*=275) were male controls from a different population-based case–control study of early-onset prostate cancer. These controls were chosen from the same general practitioners as controls in the case–control study, and were therefore recruited from across the UK. Controls had no personal history of cancer. Individuals whose ethnic group was recorded as non-white were excluded from both the case and control series.

### Lymphoblastoid cell lines

A lymphoblastoid cell line (LCL) (HA220) was established by infection with Epstein–Barr virus from a breast cancer patient carrying the *ATM* 3161G variant in the homozygous state. This individual carried none of the other SNPs investigated in this present study; however, the 2572T>C variant, which is in strong linkage disequilibrium with 3161C>G, was also present. LCLs from a subject with a wild-type *ATM* gene (IARC 1104) and a classical AT patient carrying truncating mutations on both alleles (IARC AT11 Q2002X; Q2714X) were used for comparative purposes in this study. These two lines were obtained from Dr G Lenoir. The cells were routinely maintained at 37°C in 5% CO_2_ in RPMI 1640 Glutamax-1 medium (Gibco, Invitrogen Corporation, Cergy-Pontoise, France) containing 10% heat-inactivated foetal calf serum (Integro b.v.i, Zaandan, Holland) and 1% penicillin/streptomycin (Biochrom AG, Berlin, Germany).

### Cell cycle distribution by flow cytometry

Two flasks were set-up containing 20 ml of stock cultures (5 × 10^5^ cells ml^−1^). One flask served as an unirradiated control and the second was treated with 5 Gy of ionising radiation from a Cs^137^ source. A volume of 4 ml was harvested immediately from each flask (time 0 h) and then 24 and 48 h postirradiation. The cells were centrifuged at 1100 r.p.m. for 5 min at 4°C, washed once in PBS and frozen at −80°C in citrate/sucrose/DMSO buffer (CycleTEST™ PLUS staining kit Becton Dickinson, Franklin Lakes, NJ, USA) until being evaluated. Just before analysis, the cells were resuspended in a trypsin solution for 10 min at room temperature, followed by the addition of RNAse buffer (10 min at room temperature) and stained with a solution of propidium iodide. The samples were subsequently analysed with a FACS Calibur flow cytometer (Becton Dickinson). The ModFit LT cell cycle analysis software was used to estimate percentage of cells in the G0–G1, S and G2–M phases. At least three independent experiments were done for each cell line and the G2/G1 ratios were calculated.

### DNA extraction

DNA was extracted from blood samples by routine methods with the inclusion of a second proteinase K digestion at 50°C (Edwards *et al*, 1997). DNA was dissolved in 0.2–0.4 ml of water (BDH, Poole, UK) and stored at −20°C until required.

### ATM SNP analysis

The frequency of the *ATM* SNPs was assessed using either high-performance liquid chromatography (DHPLC) or restriction fragment length polymorphism (RFLP) after polymerase chain reaction (PCR) amplification of the appropriate *ATM* fragment. For DHPLC analysis, PCRs of 40 *μ*l were performed in 96-well plates to amplify two regions using the primers listed in Table 1

**Table 1:** Primers and restriction enzymes for *ATM* exon and SNP analysis

***ATM* exon or SNP**	**Technique**	**Primers**	**Restriction enzyme and digestion conditions**
Exon 39	DHPLC	Forward: 5′-GGCAGATTAATCTATCATCTTTTAGA-3′	
		Reverse: 5′-ATTCTGTTTCATTATGGTAATGGC-3′	
		Fragment size 372 bp	
Exon 24	DHPLC	Forward: 5′-TTCATATTCAACCACAGTTC-3′	
		Reverse : 5′-TGTAAGACATTCTACTGCCATC-3′	
		Fragment size 224 bp	
3161C>G (P1054R)	RFLP	Forward: 5′-AGCACAGAAAGACATATTGGAAG-3′	o/n at 37°C 10 U *Alw*I
		Reverse: 5′-ACTATGTAAGACATTCTACTGCC-3′	
		Fragment size 494 bp	

. Each PCR contained 25 ng DNA, 200 *μ*M each dNTP, 3 mM MgCl_2_, 0.4 *μ*M primer and 1.5 U Platinum *Taq* polymerase (InVitrogen SARL, Cergy Pontoise, France). The cycling conditions were 94°C 5 min, followed by 35 cycles of 94°C 30 s, 52°C (exon 24) or 57°C (exon 39) 30 s, 72°C 30 s, with a final extension at 72°C for 5 min. The PCR products were denatured for 5 min at 95°C and then slowly cooled to permit reannealing and formation of homoduplexes and heteroduplexes.

DHPLC analysis of exon 24 (containing the 3161C>G variant) and exon 39 (containing the 5557G>A, 5558A>T, ivs38-8t>c and ivs38-15g>c variants) was performed on a WAVE DNA Fragment Analysis System (Transgenomic, Omaha, NE, USA). Buffer gradient and temperature conditions were calculated using the WAVEmaker software (version 3.4.4. Transgenomic). In order to establish whether the homoduplexes contained a wild-type or variant sequence, a second DHPLC analysis was carried out mixing these samples with a DNA sample containing a wild-type sequence. For all samples displaying aberrant DHPLC chromatograms, the PCR was repeated and the sequences of these PCR products determined using an ABI 3100 Genetic Analyser. The primers used for sequencing were the forward and reverse PCR primers in Table 1.

The frequency of the 3161C>G SNP was assessed in the DNAs of all the controls and 226 of the cases using restriction endonuclease digestion making use of the fact that this SNP occurs at a naturally occurring *Alw*I site (Table 1). The PCRs were carried out in a total volume of 25 *μ*l containing 25 ng DNA, 100 *μ*M each dNTP, 2 mM MgCl_2,_ 0.4 *μ*M primer and 1.5 U Platinum *Taq* polymerase (InVitrogen SARL, Cergy Pontoise, France). The cycling conditions were 94°C 5 min, followed by 40 cycles of 94°C 30 s, 59°C 30 s, 72°C 30 s, with a final extension at 72°C for 5 min. The PCR product was digested with *Alw*I according to the manufacturer's instructions and the fragments analysed by electrophoresis on a 3% NuSieve agarose gel. DNA samples known to be carrying the mutant allele were included in each analysis, with the genotype of the samples being determined by the banding pattern observed on the gels (the 494 bp PCR product being cut into 305 and 189 bp fragments if the wild-type allele is present). A random sample of DNAs was also analysed by direct sequencing of the corresponding exon and complete concordance between the different techniques was observed (data not presented).

### Statistical methods

Analyses of genotype frequencies for each polymorphism were based on all cases and controls successfully typed for each polymorphism. Departures from Hardy–Weinberg equilibrium were assessed by comparing the observed and expected genotype frequencies. Differences in genotype frequencies between cases and controls were tested using standard χ^2^-tests. Odds ratios (OR) and confidence limits (CI) were calculated by standard methods. For the 5557G>A polymorphism where there was more than two genotypes, 95% floating confidence limits (FCIs) were also computed as suggested by Easton *et al* (1991). An ANOVA analysis was used to compare the G2/G1 ratios between the control, AT and HA220 cell lines and a *t*-test to compare the results obtained for HA220 and the group of six control cell lines. All computations were calculated using STATA version 7.0 (Stata Corporation).

## RESULTS

The genotype frequencies for the five SNPs in cases and controls, with corresponding ORs, are shown in Table 2

**Table 2:** Association between *ATM* SNPs and prostate cancer

	**Rare allele frequency**							
**SNP**	**Controls**	**Cases**		**Cases (%)**	**Controls (%)**	**OR**	**95% CI or FCI**	***P***-**value**
5557G>A	0.166	0.151	GG	457 (72.7)	309 (69.4)	1	0.87–1.16	
			GA	153 (24.4)	124 (27.9)	0.83	0.66–1.06	
			AA	18 (2.9)	12 (2.7)	1.01	0.49–2.11	0.43
								
5558A>T	0.005	0.004	AA	623 (99.2)	440 (99)	1		
			AT	5 (0.8)	4 (1)	0.88	0.24–3.31	0.85
								
ivs38-8t>c	0.036	0.036	TT	585 (93.2)	414 (93.0)	1		
			TC	41 (6.5)	30 (6.7)			
			CC	2 (0.3)	1 (0.3)			
			C carriers	43 (6.8)	31 (7.0)	0.98	0.60–1.58	0.96
								
ivs38-15g>c	0.004	0.008	GG	618 (98.4)	441 (99.1)	1		
			GC	10 (1.6)	4 (0.9)	1.78	0.55–5.72	0.32
								
3161C>G	0.018	0.038	CC	578 (92.6)	402 (96.4)	1		
			CG	45 (7.2)	15 (3.6)			
			GG	1 (0.2)	0 (0)			
			G carriers	46 (7.4)	15 (3.6)	2.13	1.17–3.87	0.016

. The maximum number of cases and controls for whom a genotype was available was 628 and 445, respectively. The frequencies of the variant alleles 5557A, 5558T and 3161G are in good agreement with previous studies in the Caucasian population (Dork *et al*, 2001; Spurdle *et al*, 2002; Mauget-Faysse *et al*, 2003). The frequency of the intronic SNPs ivs38-15g>c (Thorstenson *et al*, 2001) and ivs38-8t>c (Sandoval *et al*, 1999) have previously only been determined in a small number of individuals. The ivs38-15 is the rarer of the two variants, the c allele being present at an allele frequency of 0.004, while the ivs38-8c allele is found at a frequency of 0.036. In agreement with previous observations, the ivs38-8t>c variant was in strong linkage disequilibrium with the G5557A variant. The allele frequency did not differ significantly between the two series of controls, or between male and female controls, for any of the SNPs examined. We therefore combined our control series for the main analysis.

We found no significant differences in the genotype distribution between cases and controls for the SNPs 5557, 5558, ivs38-8 and ivs38-15. The 3161G allele was, however, associated with an increased risk of developing prostate cancer (any G *vs* CC OR 2.13, 95% CI 1.17–3.87; *P*=0.016) (Table 2).

**Figure 1:**
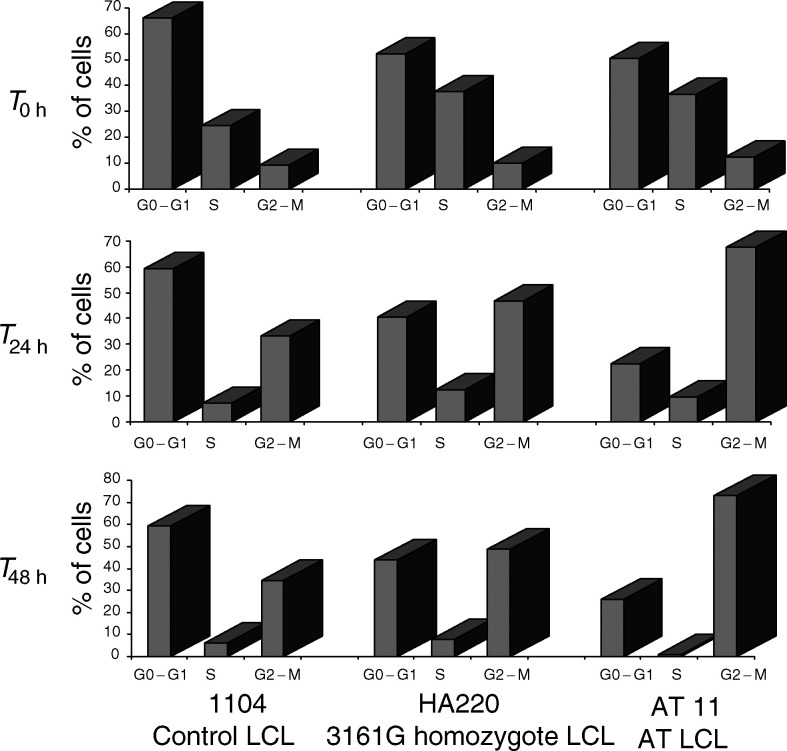
Cell cycle analysis at 0, 24 and 48 h after exposure to 5 Gy ionising radiation in LCLs carrying either a wild-type *ATM* (IARC 1104) or a mutated *ATM* (AT11) or the linked 3161G and 2572C *ATM* variants in the homozygote state (HA220).

A lymphoblastoid cell line carrying both the 3161G variant in the homozygote state and the 2572T>C variant in exon 19 (F858L), which are in strong linkage disequilibrium, was available. The line originated from a German breast cancer patient who was diagnosed at age 38 and suffered from a local relapse by age 40. Her father died from prostate cancer, but she had no family history of breast cancer. ATM protein was found to be expressed at wild-type levels in the line HA220 as revealed by immunoblotting (data not shown). The cell cycle profile after exposure to 5 Gy was determined in this lymphoblastoid line (HA220) in comparison with a LCL carrying a wild-type or a mutant *ATM* gene. At zero time (*T*_0 h_ Figure 1

) there was no differences in the proportion of cells in the different phases of the cell cycle and thus in the G2/G1 ratios between HA220, the control and AT cell lines. After exposure to 5 Gy of ionising radiation the G2/G1 ratio, representative of the percentage of cells which accumulate in G2, is significantly higher in the AT line, IARC AT11, than the control line, IARC 1104 at both 24 h (*P*=0.001) and 48 h (*P*=0.000014). The ANOVA analysis of the observed cellular response, adjusted for time, for HA220 shows that it is intermediate between that seen in these two cell types. The G2/G1 ratio was significantly higher than that seen in the control cell line after exposure (G2/G1 HA220 *vs* IARC 1104 *P*=0.033) yet lower than that seen in the AT line (G2/G1HA220 *vs* IARC AT11 *P*=0.000005). When this profile is compared with the results obtained from the analysis of six cell lines carrying a wild-type *ATM* gene assayed under similar conditions (Angèle *et al*, 2003) the response in HA220 it is not statistically different 24 h after exposure to ionising radiation. However, at 48 h postirradiation there was significantly more cells in the G2 phase of the cell cycle than in the treated control cell lines (HA220 *vs* control cell lines 48.62±3.47 *vs* 41.22±4.63% *P*=0.0296 (*t*-test) full data set not shown).

## DISCUSSION

We have investigated the possible association between five *ATM* sequence variants and an increased risk of prostate cancer. Of the SNPs investigated, we found evidence for an association with prostate cancer only for the 3161C>G (1054P>R) variant. This association, while significant at the 0.016 level, is not definitive and will require further evaluation in other case–control studies. Conversely, modest associations with some of the other SNPs cannot be definitively excluded. This is particularly true of the 5558A>T and ivs38-15g>c variants, which are rare and for which the upper 95% CI on the OR exceeds 3.

Assuming that the association between 3161G variant allele and prostate cancer is not due to chance, there are essentially three possible explanations for the association: a difference in frequency between the populations from which the cases and controls were drawn (population stratification), linkage disequilibrium or a true causal association. Although population stratification remains a possibility, the similarity in frequency between the two control groups, and the fact that one of the control groups were chosen from the general practitioners of prostate cancer cases makes this less likely. To distinguish between a causal association and linkage disequilibrium, it would be necessary to evaluate all variants occurring on the same haplotype as 3161G. Strong linkage disequilibrium has been found between 3161G (P1054R) and the variant allele at 2572C (F858L). It has also been found to occur in *cis* to the splicing mutation 3576G to A found in some AT patients of South or South East European descent (Sandoval *et al*, 1999) although this splicing mutation was neither present in the patient HA220 nor in any other breast cancer patient carrying the 3161G allele (Dork *et al*, 2001). There may, however, be other variants on this haplotype (including noncoding alterations) that have not been studied.

The 3161G>C variant is located in the *β*-adaptin domain of the ATM protein and has been suggested to be linked to an increased cancer risk (Vorechovsky *et al*, 1996, 1999). It has been reported as a pathogenic mutation in a B-cell chronic lymphocytic leukaemia patient (Stankovic *et al*, 1999). Larson *et al* (1997/8) found that the variant genotype was present in 13.6% of breast cancers with an affected sister, compared with 3.5% of breast cancers without a family history and 3.2% of the controls. The results from subsequent breast cancer studies do not provide strong support for this association (Dork *et al*, 2001; Sommer *et al*, 2002; Spurdle *et al*, 2002). A combined analysis of these three studies, however, provides some suggestion of an association between 3161G and breast cancer risk (OR after stratification by study 1.34, 95% CI 0.99–1.83). Dork *et al* (2001) found a higher proportion of node-positive tumours was observed in P1054R heterozygous breast cancer patients (P<0.01) suggesting that this *ATM* variant could modulate the course or prognosis of breast carcinoma. Interestingly, the LCL established from a homozygous carrier of the 3161G allele shows a cell cycle progression profile with time after exposure to ionising radiation that is intermediate between that seen for LCLs carrying a wild-type or a mutant *ATM* gene. In addition, this line and five other LCLs established from breast cancer patients carrying the linked 2572T>C and 3161 C>G variants in the heterozygous state had higher levels of micronuclei induction after exposure to ionising radiation compared with LCLs with a wild-type *ATM* gene (Gutiérrez-Enríquez *et al*, 2004) suggesting that the presence of this variant may influence the cellular response after exposure to ionising radiation.
